# Longitudinal Quality of Life in Adults with Metastatic Breast Cancer: The Impact of Between- and Within- Person Changes in Symptoms and Positive Affect

**DOI:** 10.21203/rs.3.rs-8138106/v1

**Published:** 2025-11-19

**Authors:** Brenna Mossman, Brent J. Small, Elizabeth L. Addington, Judith Tedlie Moskowitz, Mikaela A. Velazquez-Sosa, Jennifer D. Rodriguez, Aigner Bobbitt, Alene Mathurin, Lesley Glenn, Shontè Drakeford, Roxana Guerra, Claudine Isaacs, Ami Chitalia, Christopher Gallagher, Deena Graham, Nina Kadan-Lottick, Suzanne C. O’Neill, Claire C. Conley

**Affiliations:** Lombardi Comprehensive Cancer Center, Georgetown University, Washington, DC; School of Nursing, University of North Carolina at Chapel Hill, Chapel Hill, NC; Department of Medical Social Sciences, Feinberg School of Medicine, Northwestern University, Chicago, IL; Department of Medical Social Sciences, Feinberg School of Medicine, Northwestern University, Chicago, IL; Lombardi Comprehensive Cancer Center, Georgetown University, Washington, DC; Lombardi Comprehensive Cancer Center, Georgetown University, Washington, DC; John Theurer Cancer Center, Hackensack University Medical Center, Hackensack, NJ; John Theurer Cancer Center, Hackensack University Medical Center, Hackensack, NJ; Patient Author, Project Life Metastatic Breast Cancer, Calabasas, CA; Patient Author, Upper Marlboro, MD; Patient Author, Washington, DC; Lombardi Comprehensive Cancer Center, Georgetown University, Washington, DC; MedStar Washington Hospital Center, Washington, DC; MedStar Washington Hospital Center, Washington, DC; John Theurer Cancer Center, Hackensack University Medical Center, Hackensack, NJ; Lombardi Comprehensive Cancer Center, Georgetown University, Washington, DC; Lombardi Comprehensive Cancer Center, Georgetown University, Washington, DC; Lombardi Comprehensive Cancer Center, Georgetown University, Washington, DC

**Keywords:** ecological momentary assessment, quality of life, psychological well-being, symptom burden, breast neoplasms, metastatic cancer

## Abstract

**Introduction:**

Limited research has longitudinally assessed quality of life (QoL) among patients with metastatic breast cancer (MBC) outside of therapeutic clinical trials. This study used ecological momentary assessment (EMA) to examine QoL in daily life.

**Methods:**

Patients with MBC completed 12 EMA surveys over 4 weeks. Surveys utilized a visual analog scale (0–100) to assess global QoL, symptom severity (depression, anxiety, pain, fatigue, slowed cognitive functioning, appetite loss, nausea, gastrointestinal distress, decreased libido), and positive affect (social connection, peace, joy). Multilevel mixed-effects models analyzed the relationships between QoL and symptom severity and positive affect, when ratings differed across persons (between-person differences) and among the same person (within-person differences). We also explored longitudinal changes in QoL.

**Results:**

Participants (N=118; *M*=57.6 years old) were a mean of 4.3 years post-diagnosis. In mixed models adjusting for sociodemographic and health variables, between-subjects effects indicated QoL was worse among individuals who reported more depression (*p*=.009), more nausea (*p*=.005), and less joy (*p*<.001). Within-person effects indicated individuals’ QoL was lower when they reported worse depression (*p*<.001), anxiety (*p*=.012), and fatigue (*p*=.004), lower appetite (*p*=.019), lower libido (*p*=.018), and less social connectedness (*p*=.001), joy (*p*=.040), and peace (*p*<.001) than their average ratings. Mixed models estimated QoL was worse near diagnosis (*p*=.028), especially for patients who were older (*p*=.005) and non-partnered (*p*=.001).

**Conclusions:**

Both within-person and between-person variations in symptom severity and positive affect are associated with QoL. The relative influence of within-person variation suggests the need for adaptive, targeted intervention strategies to optimize patients’ quality of life.

## Introduction

Treatment advances in metastatic breast cancer (MBC) have significantly increased patients’ survival time following diagnosis.^1–4^ In 1990, the median survival following MBC diagnosis was 14–32 months; as of 2010, it has nearly doubled to 31–57 months.^4^ These lengthening survival times have contributed to a growing population of individuals living with MBC. Nearly a quarter of a million people in the United States will be living with MBC by 2030, almost a 60% increase from 2017.^5,6^ As individuals live longer with this incurable condition, understanding and supporting quality of life (QoL) is a priority.^7^ MBC can entail significant physical and psychological symptoms that limit daily functioning and activities, yet this population is often excluded from cancer survivorship studies supporting QoL.^8–10^ Moreover, little information exists regarding longitudinal changes in these outcomes across the cancer trajectory.^11^ As such, further research is needed to understand patients’ everyday experiences underlying their QoL, including how these experiences may evolve from the time of diagnosis.

Prior research examining QoL longitudinally among those with MBC is primarily restricted to pharmaceutical clinical trials.^11–15^ While valuable, such studies may not be reflective of the broader population, as they enroll < 10% of affected patients,^16^ and their inclusion criteria often requires higher functional statuses and excludes those with significant comorbidities.^17,18^ These trials’ assessment timepoints also typically align with the beginning and end of treatment cycles, overlooking more granular changes that happen within treatment and reducing generalizability to QoL between treatment cycles. Evidence outside of pharmaceutical trials, while limited, offers conflicting results.^19,20^ Whereas data from pharmaceutical trials have primarily found significant decrements to QoL over time,^13–15^ recent observational studies have reported no or minimal changes, emphasizing a need for further research.^19–21^ Additionally, existing longitudinal assessments of QoL typically employ retrospective reports and relatively long periods between study assessments, which may obscure important fluctuations in patients’ everyday symptom experiences that inform QoL.^22^ Thus, existing research may not adequately reflect the real-world daily experiences of those living with MBC, and patterns of QoL over time in this population require further clarification.

To address these gaps, the present study assessed patterns of QoL among individuals with MBC using ecological momentary assessment (EMA), a methodological approach that can improve ecological validity and capture dynamic patterns of change.^22–24^ EMA asks participants to repeatedly report their experiences in the present moment and therefore provides rich, temporally-sensitive data that offers insight into participants’ well-being between typical monitoring appointments.^22–24^ Using this EMA data, the present study sought to understand changes in QoL that may occur between and within individuals, as well as over time. Specifically, the study aimed to examine the relationships between QoL and differences in symptom burden and positive affect across different individuals (between-person differences) and among the same individual over days of assessment (within-person differences). We also explored longitudinal changes in QoL over time since MBC diagnosis.

## Materials & Methods

This longitudinal, observational study among people living with MBC utilized EMA to examine QoL (Georgetown University Institutional Review Board study #00005081). This research was designed and conducted with the active input of patient advocate collaborators to ensure the methodology was informed by the perspectives of individuals living with MBC.

### Participants and procedures

Participants were recruited at five sites: 1) MedStar Georgetown University Hospital (Washington, DC), 2) MedStar Washington Hospital Center (Washington, DC), 3) MedStar Good Samaritan Hospital (Baltimore, MD), 4) MedStar Franklin Square Medical Center (Baltimore, MD), and 5) Hackensack Meridian Health John Theurer Cancer Center (Hackensack, NJ). Participants met the following inclusion criteria: receiving care at one of the five sites for a diagnosis of metastatic breast cancer, assigned female at birth, current age ≥ 18, English- or Spanish-speaking, and possession of working cell phone with SMS messaging and internet-access. We restricted the sample to participants assigned female at birth due to the rare and distinct nature of breast cancer in individuals assigned male at birth.^25^ We excluded individuals who were unable to complete study assessments on their own and those who had a life expectancy of less than 6 months as determined by their treating breast oncologist.^26^

After providing informed consent, participants completed a baseline survey pertaining to sociodemographic and health characteristics. Participants also underwent an EMA “training” session to ensure functionality and understanding of the process. Following this training, participants began completing EMA prompts, which consisted of timed text reminders to complete assessments of global QoL, symptom severity, and positive affect (see supplemental materials). EMA prompts occurred as bursts: three times a day (morning, afternoon, evening), one day per week (randomly assigned each week), for four consecutive weeks. This “measurement burst” design incorporates short periods of intensive, recurrent assessment that are repeated longitudinally and spaced out over longer intervals. This design reduces participant burden while maintaining density of assessments.^27,28^ On average, participants completed the assessments in 2 minutes and 47 seconds. Participants received up to $35 for completing all surveys.

## Measures

### Sociodemographic and health characteristics

Participants self-reported their age, gender identity, race, ethnicity, relationship status, sexual orientation, education, employment, income, insurance status, and current caregiving responsibilities (yes/no). Clinical characteristics were abstracted from electronic medical records, including breast cancer subtype (hormone receptor, HER2 status) and treatment type(s).

### Ecological momentary assessments: QoL, symptom severity, positive affect

Participants reported their global QoL, symptom severity, and positive affect using a single visual analog scale item.^29,30^ Response options ranged from 0 to 100, with higher scores indicating better QoL, greater severity of symptoms, and greater positive affect. The global QoL item asked participants, “how would you rate your quality of life right now?,” describing QoL as overall enjoyment of life, including physical, emotional, and social well-being (see supplemental materials). Symptom severity items included current severity of depression, anxiety, pain, fatigue, slowed cognitive functioning, appetite loss, nausea, gastrointestinal (GI) distress, and decreased libido. Positive affect items included current experiences of social connectedness, peace, and joy, which were selected in collaboration with patient advocates based on their relevance to the experiences of patients with MBC. Single visual analog scale items have been used extensively to assess quality of life among individuals with cancer and demonstrate good reliability and validity.^31,32^

### Analyses

All analyses were conducted using IBM SPSS Statistics for Windows, version 28 (IBM Corp., Armonk, N.Y., USA). Descriptive statistics were used to characterize the sample. Bivariate Pearson correlations were then used to examine the associations between baseline sociodemographic and health variables and QoL (for all observations across all timepoints). Sociodemographic and health variables significantly associated with QoL at *p* < .05 were included within the multilevel mixed models as covariates.

**Figure 1:**
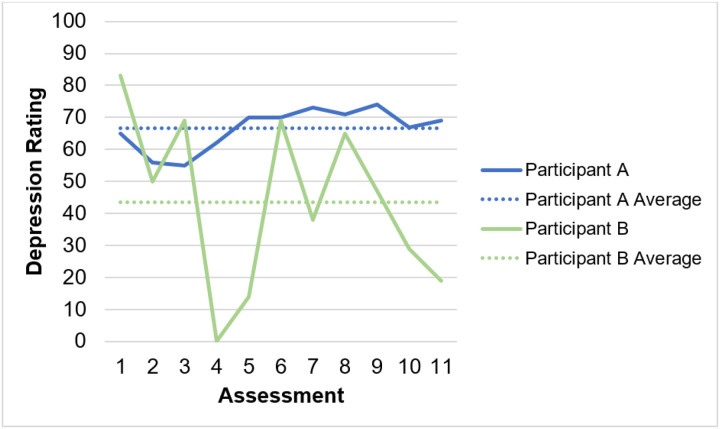
Examples of within- vs. between-person variation: Depression ratings for two participants across study assessments *Note*. Figure depicts patterns of depression among two participants. Participant A has a higher overall average rating of depression, but less within-person variability in ratings. Participant B has a lower overall average rating, but more within-person variation.

We employed mixed models to examine predictors and variations in QoL over time. First, we evaluated the extent to which QoL, symptoms, and positive affect varied within and between persons. Then, we used multilevel models to assess whether symptom and positive affect experiences predicted QoL using both within- and between-person estimates of each predictor. That is, each symptom and positive affect construct had a corresponding within-person variable (i.e., deviation from an individual’s average; person-mean centered), as well as a between-person variable (i.e., deviation from the group’s average; grand mean centered). This allowed the models to assess, for example, the impact of times when an individual feels more or less depressed than their personal average, as well as the impact of an individual being, on average, more or less depressed than the sample’s mean level (see Fig. 1 for an illustration of these within- vs. between-person effects). Finally, we used growth models to explore longitudinal changes in QoL across years since diagnosis. We did not follow participants from the time of diagnosis, but rather utilized time since diagnosis as a meaningful time variable within the context of this study to estimate change over time. We utilized conditional growth models, adjusting for significantly correlated sociodemographic and health variables, to assess whether quality of life and its changes over time since diagnosis were related to sociodemographic and health characteristics.

Based on the prior literature, *a priori* power analyses assumed 10% attrition at each measurement burst and an EMA response rate of 78–88% of EMA prompts, resulting in an anticipated 964–1,087 observations over the course of the study. Analyses indicated that the study would be powered at > 91% to detect a small-to-medium fixed effect of d = 0.25 at α = .05 and a person-level intraclass correlation coefficient (ICC) of .05. The study’s final observation count (i.e., 1,101) exceeded this hypothesized amount and therefore met this aim.

## Results

**Figure 2:**
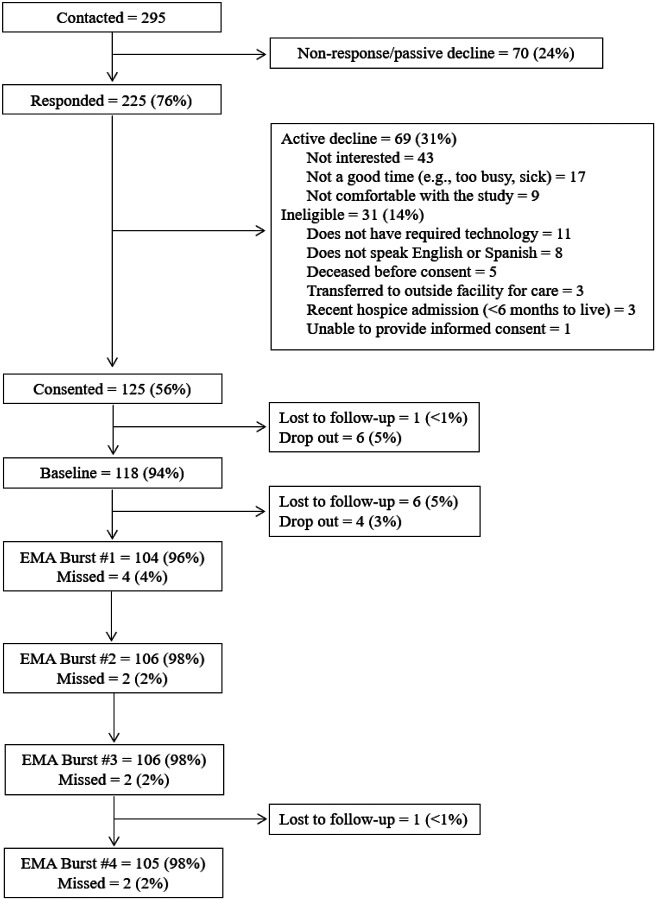
Study Participant Flow *Note*. Lost to follow up refers to individuals who became unresponsive to study team contact. Drop out refers to individuals who confirmed they no longer wished to participate in the study.

A total of 295 patients were contacted, and 225 responded (Figure 2). Of those who responded, 125 consented (56%). The remainder either actively declined (31%), or were ineligible (14%). Reasons for ineligibility included not having the required technology, not speaking English or Spanish, dying before consent, moving to an outside care facility or hospice care and therefore having <6 months to live, or being unable to provide informed consent (see Figure 2). A total of 118 participants comprised the final baseline sample, and loss to follow up and dropout remained below 10% throughout EMA bursts (total drop out of 8% from time of consent). The final amount of observations totaled 1,101, with an average cluster size of 10.2 observations per participant (range: 1–12).

### Sample characteristics

Table 1 presents the sample’s sociodemographic and health characteristics. Participants were, on average, 57.6 years of age and diagnosed 4.3 years ago (range: 0.06 – 20.65 years; median=2.5 years). Participants most frequently identified as Non-Hispanic White (47.5%), Non-Hispanic Black (35.6%), or Hispanic or Latino/a (10.2%).

A number of demographic characteristics were significantly associated with QoL ratings across time points (see Table 1). Patients who were older (r=.100, *p*=.001), Non-Hispanic/Latino/a (v. Hispanic/Latino/a, r=-.174, *p*<.001), Middle Eastern or North African (v. not, r=.084, *p*=.006), Native Hawaiian or Pacific Islander (v. not, r=.088, *p*=.004), partnered (v. not, r=.248, *p*<.001), with a higher annual household income (r=.081, *p*=.014), and private health insurance (v. not r=.122, *p*<.001) reported higher QoL across time points. Among health characteristics, participants who were closer to the time of diagnosis (r=-.094, *p*=.002), with HER2-negative (v. positive, r=-.068, *p*=.025) or hormone receptor (HR)-positive disease (v. negative, r=.095, *p*=.002), who did not receive radiation (r=-.088, *p*=.004) or immunotherapy during the study period (r=-.089, *p*=.003), and who received hormone therapy during the study period (r=.081, *p*=.008) reported higher QoL. These variables were included within the multilevel mixed models as covariates.

### QoL, symptom burden, and positive affect

Across all EMA timepoints, participants had a mean overall QoL rating of 71.1 (see Table 2). On average, participants’ most severe symptoms were decreased libido (59.5) and fatigue (35.8), while appetite loss (21.2) and nausea (10.7) were least severe. Unconditional no growth models indicated that between-person variation explained 72.2% of the variance in QoL scores. Among symptoms and positive affect, the amount of between-person variation was highest among libido (89.9%), cognition (76.0%), and social connection (74.8%). All symptom severity and positive affect scores were significantly correlated with QoL ratings at *p*<.001 (*r* values ranging from ±.340−.776). See Table 2 for full descriptive characteristics, ICCs, and correlation coefficients.

### Between- vs. within-person predictors of QoL

Mixed models (Table 3) indicated that, among between-person predictors, quality of life was worse among individuals who reported more depression (β= −.180, *p*=.009), and more nausea (β= −.138, *p*=.005), and better among those who reported more joy (β= .625, *p*<.001). Additionally, within-person predictors indicated that QoL was lower at times when individuals had more depression (β= −.169, *p*<.001), anxiety (β= −.052, *p*=.012), and fatigue (β= −.054, *p*=.004), as well as lower appetite (β= −.053, *p*=.019) and libido (β= −.063, *p*=.018) compared to their personal average. QoL was higher when individuals had more social connectedness (β= .086, *p*=.001), joy (β= .054, *p*=.040), and peace (β= .114, *p*<.001) compared to their personal average.

### Changes in QoL over time

Unconditional growth models did not find significant linear (*p*=.743) or quadratic (*p*=.414) changes in QoL over time. We then examined conditional growth models including sociodemographic and clinical characteristics (Table 4; clinical characteristics were not significantly related and therefore not included within this final model). Compared to the unconditional growth model, the conditional growth model presented in Table 4 demonstrated better model fit (change in −2LL=163.47; AIC=147.47; BIC=107.82). Participants who were younger in age (β=.516, *p*=.005) and non-partnered (β=15.093, *p*=.001) had significantly lower QoL at diagnosis than those who were older or partnered. When controlling for the effects of age, partner status, Hispanic/Latino/a ethnicity, and private health insurance, QoL was significantly positively related to time since MBC diagnosis (β=5.411, *p*=.028).

## Discussion

This prospective, longitudinal study of 125 women with MBC examined QoL by within- and between-person variations in symptom severity and positive affect, as well as its estimated change over time. The results indicated that both within- and between- person variation in symptoms and positive affect were important determinants of individuals’ QoL, which was estimated as worst near the time of diagnosis. These results have important implications for delivering *timely* interventions and clinical care that are *tailored* to individuals living with MBC.

To our knowledge, this study is the first to compare the unique influences of between- vs. within-person symptom variation on overall QoL among individuals with MBC. While both within- and between-person differences predicted QoL, the pattern of results emphasize the importance of within-person predictors. Instances when individuals are feeling worse than usual may be particularly influential in determining their QoL, and these instances may therefore be ideal times to provide intervention.^33^ This concept can be understood as an adaptive or “just-in-time” intervention, in which dynamic symptom monitoring can effectively identify when patients need more support and deliver the type and amount of support needed, accordingly. This would allow for intervention not only at the time of diagnosis, when we observed the overall poorest QoL, but also over time when indicated by symptom reporting. Existing interventions to improve QoL in those living with MBC are sparse and demonstrate varying efficacy, leaving patients with unmet supportive care needs.^8–10,34–36^ Utilizing an adaptive approach may help interventions better reach vulnerable patients and provide tailored, timely support that addresses these unmet needs.

Moreover, the pattern of significant symptoms and positive affect experiences present important considerations for research and practice. Overall, the physical symptoms significantly predicting patients’ QoL (particularly fatigue, appetite, and libido) may be related to the significant psychosocial experiences (depression, anxiety, and lower positive affect). Prior research in breast cancer has suggested the existence of symptom clusters that include combinations of these psychosocial and physical symptoms.^37^ It may be useful to explore how these experiences may cluster specifically within MBC to inform clinical practice. For example, patients with MBC may benefit from expanded supportive care options to address these symptoms more comprehensively, such as nutrition consults, sexual health focused interventions, and mental health and social support resources.

Similarly, the pattern of findings highlights the importance of positive affective experiences in patients’ QoL. Individuals’ levels of social connection, peace, and joy all significantly predicted overall QoL, yet existing interventions to improve psychosocial well-being in this population primarily focus on reducing negative affect rather than boosting positive affect.^10,36^ Specifically, only one positive psychological intervention has been conducted among patients with metastatic breast cancer, and it was completed nearly 10 years ago.^38^ Considering advances in treatment and individuals’ changing experiences living with MBC, additional research is required to further explore the impact of positive affect on QoL and the utilization of positive affect as a target of intervention.

Additionally, results demonstrated that patients’ levels of QoL at diagnosis varied with sociodemographic characteristics. Specifically, QoL estimates were lower at diagnosis for those who were younger and non-partnered. These findings build upon previous research suggesting a significant need for support among these individuals: unmarried and younger patients may present with more aggressive MBC subtypes, be diagnosed at later stages, and experience high levels of psychosocial distress during and after treatment.^39–44^ Together, these findings emphasize the need for additional, tailored support for younger and/or non-partnered patients by the clinical team, particularly near diagnosis.

Finally, we explored changes in QoL over time since MBC diagnosis. We initially observed no relationship between time since diagnosis and QoL. When controlling for sociodemographic covariates, we did observe a relationship between QoL and time since diagnosis, such that QoL was worst near the time of diagnosis. This was a surprising finding, and not consistent with the lived experiences of our patient advocate collaborators. These longitudinal findings must be interpreted with caution for several reasons. First, we did not follow a cohort of patients with MBC from the time of diagnosis. Rather, we recruited patients who had been living with MBC for any period of time. As a result, our sample had a very wide range of years since MBC diagnosis (range: 0.06–20.65 years). Our statistical analyses modeled change in QoL over time since diagnosis because it was more meaningful and interpretable than time in the study. However, the trends observed here are statistical projections and may differ from QoL data from long-term longitudinal observational studies. Additionally, significant change in QoL was only seen after adjusting for covariates. Lastly, the findings reflect the experiences of those still able and willing to participate in this research, who may have relatively higher levels of QoL than non-participants. Nonetheless, these findings represent an interesting and counterintuitive pattern that should be examined further in longitudinal cohort studies of patients living with MBC.

### Strengths and limitations

The present study contains a number of methodological strengths. The sample had a diverse representation of race and ethnicity, MBC subtypes, treatments, and time since diagnosis. The use of EMA allowed for frequent collection of data to provide a representative depiction of daily life with MBC, and the measurement burst design was feasible and acceptable for participants, with high completion rates across bursts (≥ 96%). This methodology yielded unique data that few other studies have collected in women with MBC. Furthermore, the methodology was designed in collaboration with patient advocates, ensuring the assessments’ relevance to patients’ lived experiences.

The study also has several potential limitations. Of the 225 individuals we established contact with, 56% consented to participate; we do not have data on non-responders or those who declined, so we are not able to compare, but it is possible that the results may be subject to selection bias. Participants also self-reported their experiences using single visual analog scale items, which may be susceptible to social desirability and demand characteristics (i.e., reports that are influenced by a desire to align with expectations or social norms). Additionally, while the use of single items reduced participant burden, they may not perform as well as multi-item measures of QoL or symptom burden; however, prior research has established their relative equivalence with regard to psychometric performance.^31,32^ Finally, there is still unexplained variance within the models (i.e., as assessed via pseudo R^2^ measures), suggesting there are important but unmeasured variables that may further predict QoL. We chose to focus on this study’s symptoms and positive affect due to their relevance to our patient collaborators’ lived experiences, but other constructs, such as spiritual or socioeconomic factors, may contribute to quality of life, as well.^45^

## Conclusion

Given that treatment advances are lengthening the survival of patients diagnosed with MBC, additional research is needed to understand and support the QoL of these patients. The present study found substantial within-person and between-person variations in symptom severity and positive affect that were associated with QoL, and results suggest that within-person variation in symptoms and positive affect are important but understudied determinants of QoL. QoL also varied by sociodemographic factors and time since MBC diagnosis. These data highlight the need for adaptive, targeted intervention strategies to optimize quality of life for all patients.

## Figures and Tables

**Table 1.** Sociodemographic and health characteristics (N=118)

Characteristic	N (%) or Mean (SD)	Correlation with quality of life
Age (years)	57.6 (12.2)	.100*
Race and ethnicity^a^		
American Indian or Alaska Native	1 (0.8%)	−.037
Asian	9 (7.6%)	−.035
Black	42 (35.6%)	.056
Hispanic or Latino/a	12 (10.2%)	−.174**
Middle Eastern or North African	2 (1.7%)	.084*
Native Hawaiian or Pacific Islander	1 (0.8%)	.088*
White	58 (49.2%)	.009
Relationship status		
Partnered	62 (52.5%)	.248**
Single	53 (44.9%)	
Missing	3 (2.5%)	
Sexual or gender minority (SGM) status		
Yes	5 (4.2%)	-.034
No	108 (91.5%)	
Missing	5 (4.2%)	
Education level		
Less than Bachelor's degree	51 (43.2%)	-.037
Bachelor's degree or higher	67 (56.8%)	
Employment status		
Employed	58 (49.2%)	.053
Unemployed	50 (42.4%)	
Missing	10 (8.5%)	
Annual household income (USD)		
<$15,000	10 (8.5%)	.081*
$15,000–24,999	8 (6.8%)	
$25,000–49,999	20 (16.9%)	
$50,000–75,999	9 (7.6%)	
$75,000–99,999	6 (5.1%)	
≥$100,000	47 (39.8%)	
Missing	18 (15.3%)	
Health insurance status^a^		
Private	80 (67.8%)	.122**
Public	56 (47.5%)	−.052
Current caregiving responsibilities		
Yes	40 (33.9%)	-.028
No	77 (65.3%)	
Missing	1 (0.8%)	
Time since diagnosis (years)	4.3 (4.4)	−.094*
Metastatic breast cancer subtype^a^		
HER2 status (positive)	32 (27.1%)	−.068*
HR status (positive)	87 (73.7%)	.095*
Current treatment(s)^a^		
Chemotherapy	34 (28.8%)	.009
Radiation	7 (5.9%)	−.088*
Targeted therapy	70 (59.3%)	−.023
Immunotherapy	16 (13.6%)	−.089*
Hormone therapy	66 (55.9%)	.081*
Treatment changed during study	23 (19.5%)	−.016

*Note*. SD=standard deviation; HR=hormone receptor.

* p<.05;

** p<.001

a Responses not mutually exclusive; counts reflect all response options that applied. Totals may therefore not equal 100%.

**Table 2.** Quality of life descriptive characteristics

Characteristic	Range	Mean (SE)	ICC	Correlation with quality of life*
Global quality of life	15–100	71.1 (1.6)	.722	--
Symptom severity				
Depression	0–96	25.7 (2.0)	.694	−.750
Anxiety	0–100	30.9 (2.1)	.663	−.636
Pain	0–100	23.3 (2.1)	.702	−.342
Fatigue	0–100	35.8 (2.2)	.646	−.542
Slowed cognition	0–98	24.5 (1.9)	.760	−.573
Appetite loss	0–95	21.2 (2.0)	.678	−.377
Nausea	0–99	10.7 (1.4)	.539	−.367
Gastrointestinal distress	0–99	24.1 (1.9)	.542	−.493
Decreased libido	0–100	59.5 (3.2)	.899	−.340
Positive affect				
Social connection	0–100	75.3 (1.9)	.748	.709
Peace	0–100	73.2 (1.9)	.703	.766
Joy	0–100	70.1 (1.8)	.660	.776

*Note*. SE=standard error of the coefficient; ICC=intra-class correlation coefficient. Higher scores indicate better quality of life and more symptom severity and positive affect as assessed by visual analog scale items (possible score range 0–100). Mean and standard error of the coefficient obtained via unconditional no growth models.

* All correlations significant at *p*<.001

**Table 3.** Multilevel model results of between- and within-person predictors of global quality of life

Predictor	β	SE	*p*
Between-person			
Depression	−.180	.067	.009
Anxiety	−.003	.043	.950
Fatigue	−.052	.034	.132
Appetite	.012	.036	.745
Nausea	−.138	.048	.005
Libido	−.009	.017	.573
Social connection	.064	.050	.204
Joy	.625	.081	<.001
Peace	−.124	.101	.221
Within-person			
Depression	−.169	.025	<.001
Anxiety	−.052	.020	.012
Fatigue	−.054	.019	.004
Appetite	−.053	.023	.019
Nausea	−.028	.023	.214
Libido	−.063	.027	.018
Social connection	.086	.027	.001
Joy	.054	.026	.040
Peace	.114	.028	<.001
Private insurance	−2.137	1.197	.077
Intercept	55.686	3.762	<.001
Variance components			
Intercept	18.382	3.586	<.001
Residual	70.989	3.296	<.001

*Note*. SE=standard error. Quality of life was assessed via visual analog scale (possible score range 0–100; higher scores indicate better quality of life). Conditional pseudo R^2^ = .799.

**Table 4.** Multilevel model results of sociodemographic predictors of change in quality of life over time

Predictor	β	SE	*p*
Fixed effects - Intercept			
Time since diagnosis (years)	5.411	2.391	.028
Age	.516	.176	.005
Hispanic or Latino/a	−14.022	7.892	.079
Relationship status	15.093	4.575	.001
Private insurance	8.327	4.604	.075
Fixed effects - Slope			
Time*Age	−.067	.036	.068
Time*Hispanic or Latino/a	.893	1.264	.483
Time*Relationship status	−.988	.774	.206
Time*Private insurance	−1.346	.885	.133
Intercept	28.934	11.939	.018
Variance components			
Intercept	235.841	62.341	<.001
Residual	103.121	4.718	<.001

*Note*. SE=standard error. Quality of life was assessed via visual analog scale (possible score range 0–100; higher scores indicate better quality of life). Conditional pseudo R^2^ = .713.

## Data Availability

The data that support the findings of this study are openly available on the Open Science Framework at http://doi.org/10.17605/OSF.IO/RYZKD.
